# Infrahepatic caudal/inferior vena cava interruption with azygos/hemiazygos continuation. Vascular anomaly in swine

**DOI:** 10.2478/v10019-010-0029-5

**Published:** 2010-09-09

**Authors:** Miran Jeromel, Dusan Pavcnik

**Affiliations:** 1 Dotter Interventional Institute, Oregon Health Sciences University, Portland, OR, USA; 2 Institute of Radiology, University Clinical Center, Ljubljana, Slovenia

**Keywords:** experimental animal model, congenital vascular anomaly, azygos vein, hemiazygos vein, inferior vena cava

## Abstract

**Background:**

Swine are commonly used as a model to study congenital cardiovascular defects that occur in humans and these models have been both spontaneous and experimentally induced. Ventricular septal defect, patent ductus arteriosus, and atrial septal defect (ASD) are examples of experimentally induced models. Absence of caudal/inferior vena cava (CVC/IVC) with azygos/hemiazygos continuation is an uncommon vascular anomaly.

**Case report:**

The vascular anomaly presented in this case report was an incidental finding on a pig that was evaluated for experimental percutaneous atrial septal defect creation and its closure using a percutaneous femoral vein approach. Absence of CVC/IVC was confirmed by venography and necropsy.

**Conclusions:**

To the best of the investigators knowledge, this is the first report of absence of CVC/IVC with azygos/hemiazygos continuation in the swine.

## Introduction

Swine and ovine are frequent used for the experimental studies of interventional radiology procedures.^1^,^2^ However, swine are commonly used as a model to study congenital cardiovascular defects that occur in humans and these models have been both spontaneous and experimentally induced. Ventricular septal defect, patent ductus arteriosus, and atrial septal defect (ASD) are examples of experimentally induced models. In necropsy surveys of commercial breeds of farm pigs, ASD was detected in 31/1906 pigs for an incidence of 1.6%.^1^ Swine have been used as a model to produce a functional ASD by using a transeptal stationary angioplasty balloon technique.^1^

Caudal vena cava (CVC) in animals is the equivalent of inferior vena cava (IVC) in humans and normally, CVC/IVC provides the main channel of drainage for the hind limbs, abdominal muscles, and abdominal organs through the portal and hepatic veins. The main tributaries of the CVC/IVC are common iliac, lumbar, deep circumflex iliac, right testicular or right ovarian, renal phrenicoabdominal, hepatic, and phrenic veins.^3^,^4^,^5^

The vascular anomaly presented in this case report was an incidental finding on a pig that was evaluated for experimental percutaneous atrial septal defect creation and its closure using a percutaneous femoral vein approach. To the best of the investigators knowledge, this is the first report of absence of CVC/IVC with azygos/hemiazygos continuation in the swine.

## Case report

The study protocol was approved by the Oregon Health & Science University’s (OHSU) Animal Care and Use Committee (IACUC). The animal facilities are accredited by the American Association for the Accreditation of Laboratory Animal Care international (AAALAC international) and meet all federal (AWA and PHS) guidelines for animal care. The animal room was maintained at an average temperature of 68º F and a relative humidity of 30–70%. A female domestic swine (*Sus scrofa domestica*), 38 kg of body weight and approximately 4 months of age, was evaluated for experimental transcatheter implantation of a closure device for foramen ovale, using the percutaneous femoral vein approach. The swine was acclimated for at least 48 h before the terminal procedure.

Preanesthesia treatment included 0.01 mg/kg of atropine sulfate (American Regent Laboratories, Shirley, NY, USA) and 1 g dose of Cephasolin (Ancef; Abbot Laboratories, Chicago, IL, USA) intramuscularly. Anesthesia was induced with Telazol (tiletamine HCI and zolazepam HCI; Fort Dodge Animal Health, Fort Dodge, IA) 3–6 mg/kg, IM, and an endotracheal tube was placed. Maintenance of anesthesia was done with 2–3% isoflurane (Isothesia, Burns Veterinary Supply, Rockville Center, NY, USA). During anesthesia, oxygen, carbon dioxide, EKG, respiration and heart rate were monitored, and a GE/OEC 9800 cardiac mobile system with digital imaging (GE Medical Systems, OEC, Salt Lake City, UT) was used for imaging.

A size 7 French vascular sheath (Cook Inc., Bloomington, IN, USA) was percutaneously introduced into right femoral vein, and then a guide wire and a size 5 French catheter (Cook Inc., Bloomington, IN, USA) were inserted into right femoral vein and advanced cranially. After fluoroscopy showed the catheter located on the left side of the spine, contrast medium (Hypaque-76, Amersham, Piscataway, NJ, USA) was injected to perform a venogram for evaluation of venous anatomy. In addition, two size 7 French vascular sheaths were percutaneously introduced, one into the right femoral artery and the other into the right jugular vein for performance of bilateral renal artery angiograms and a jugular venogram, respectively. The right hepatic vein was then catheterized and visualized from the right jugular approach using a size 5 French H1 catheter (Cook Inc., Bloomington, IN, USA) passed through the right atrium. At the end of the procedure the animal was euthanized while under anesthesia with an overdose of sodium pentobarbital (Euthasol: Delmarva Lab, Midlothian, VA, USA).

## Diagnostic findings

**FIGURE 1:**
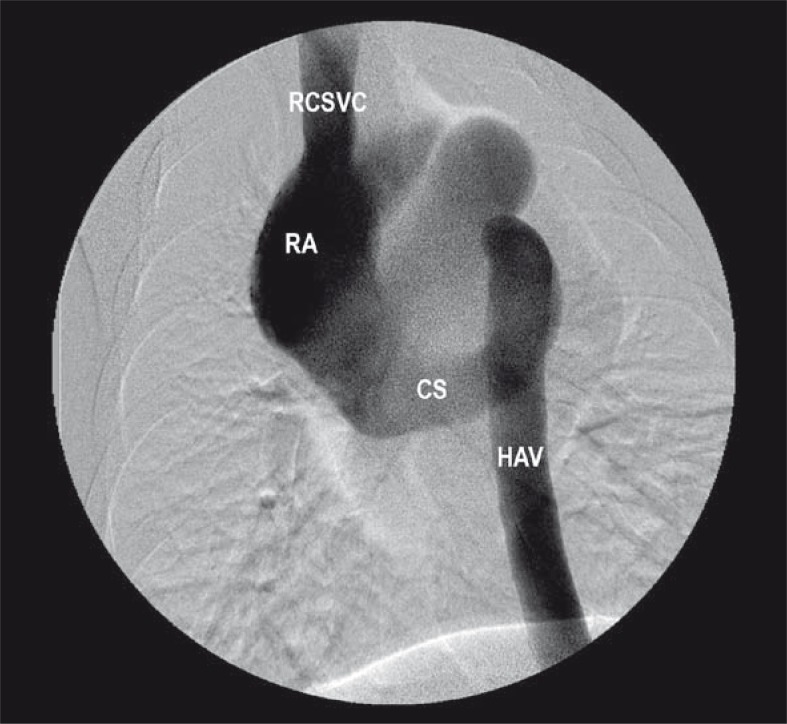
Ventrodorsal subtraction venogram of the chest in a swine after simultaneous contrast injection into the right jugular vein and right femoral veins. Injection into the right femoral vein demonstrates the large hemiazygos trunks (HAV) draining into the coronary sinus (CS), which then communicates directly with the right atrium (RA). Injection into the right jugular vein shows normal right cranial/superior vena cava (RCSVC) draining into right atrium.

**FIGURE 2:**
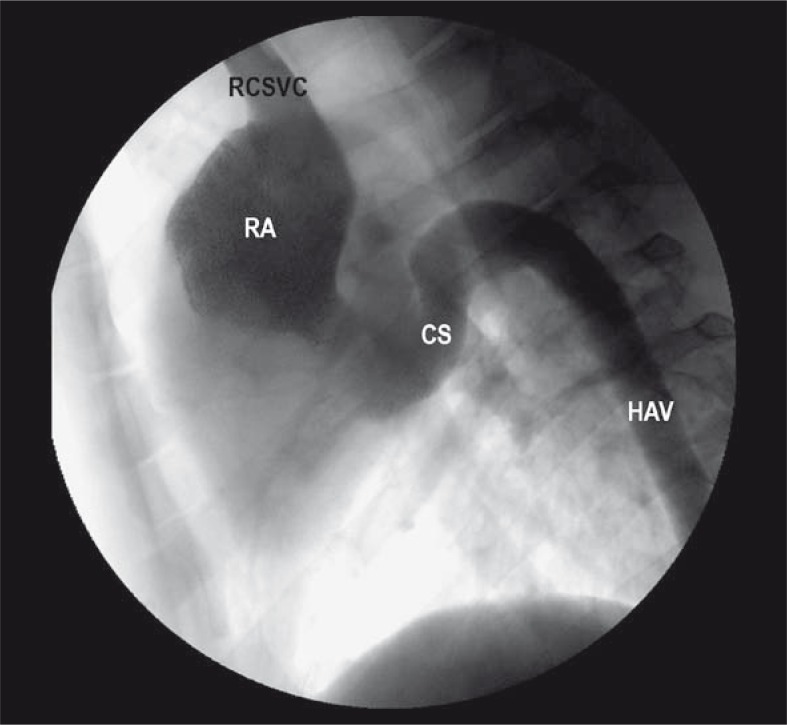
The lateral venogram of the chest in a swine after simultaneous injection of the right femoral vein and right jugular veins. The large hemiazygos vein (HAV) ascends along the vertebral column and joins the coronary sinus (CS), which then communicates the directly with the right atrium.

The ventrodorsal subtraction venogram after simultaneous injection via the right femoral vein and the right jugular vein is shown in Figure 1. Normal superior or cranial vena cava was seen on the right side, but CVC/IVC was not observed. A dilated hemiazygos vein was seen on the left side of the lumbar vertebra, emptying into the coronary sinus, which communicated directly with the right atrium. The lateral venogram after simultaneous injection of the right femoral and right jugular vein shown in Figure 2 demonstrated anomalous drainage of blood from the hemiazygos vein that abnormally emptied into the coronary sinus and then into the right atrium.

**FIGURE 3:**
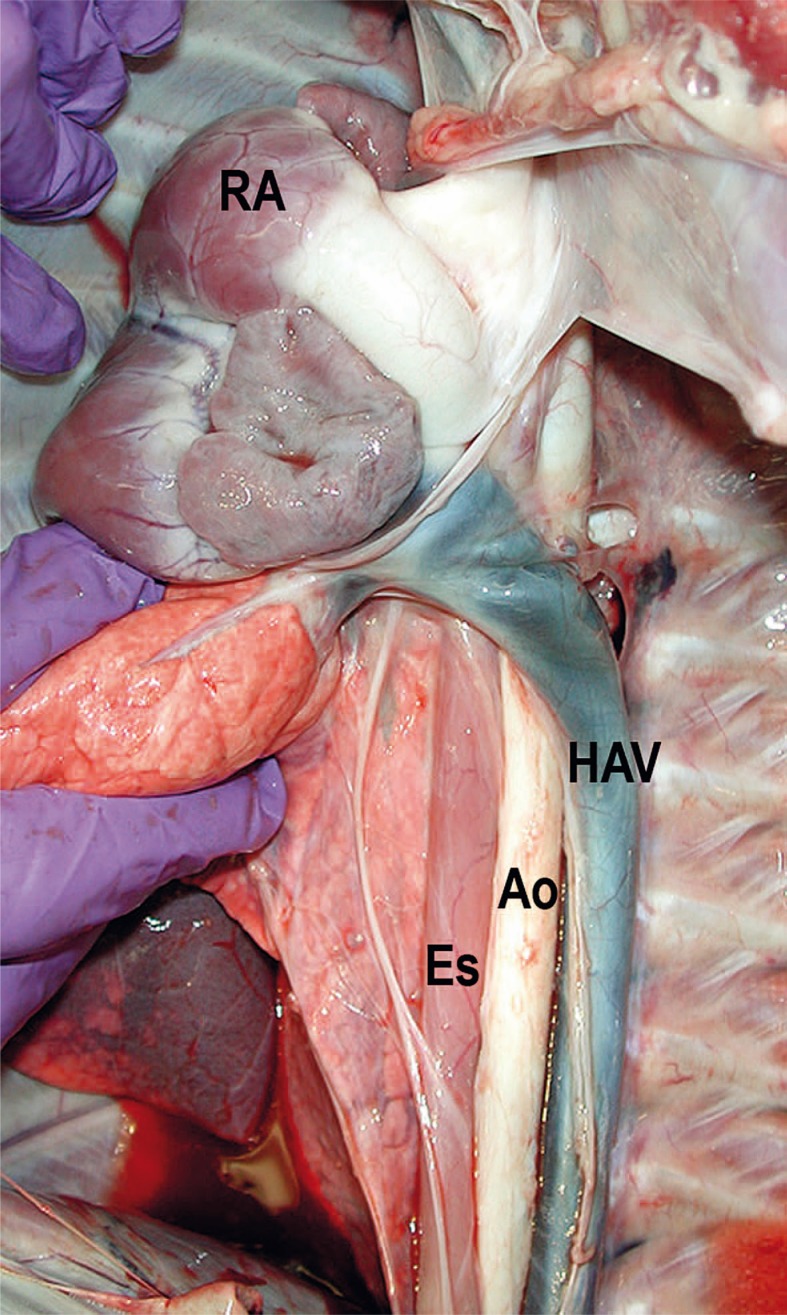
Gross specimen of the mediastinum demonstrates entrance of the hemiazygos vein (HAV) into right atrium (RA). The large hemiazygos vein drains into the right atrium across the aorta (Ao) and esophagus (Es).

The lateral subtraction venogram after simultaneous injection of contrast via right femoral vein and right hepatic vein identified the hepatic vein as the only drainage into the suprahepatic CVC/IVC. The infrahepatic CVC/IVC was not seen or identified during the venogram. The hemiazygos vein drainage into the coronary sinus was the major channel from the abdomen. Both renal veins seen on late images of renal arterigrams drained into the azygos and hemiazygos chains. The abnormality was diagnosed as infrahepatic CVC/IVC interruption with azygos/hemiazygos continuation. This finding of infrahepatic CVC/IVC interruption with azygos/hemiazygos continuation was confirmed at necropsy (Figure 3). Because of the found anomaly, the planed percutaneous creation of ASD using a femoral approach could not be performed.

## Discussion

Anomalies of the CVC/IVC are often associated with congenital heart disease. Its prevalence is 0.6–2.0% in patients with congenital heart disease and less than 0.3% among otherwise normal patients.^6^ During embryogenesis, the IVC/CVC is made up of the hepatic, prerenal, renal, post renal segments, which by segmental fusion, regression, and mid-line anastomosis form the CVC/IVC.^7^ Failure of fusion between the hepatic and prerenal segments results in infrahepatic CVC/IVC interruption that is the most common developmental anomaly of CVC/IVC.^8^ The infrahepatic CVC/IVC may continue as the azygos vein^7^,^9^,^10^, or it may continue as the hemiazygos vein to the persistent left superior vena cava^10^, intrathoracic veins^11^, or anomalous intrahepatic veins.^12^ Infrahepatic CVC/IVC interruption with azygos and hemiazygos continuation is associated with congenital cardiac or visceral malformation in the human.^5^,^8^

Segmental lumbar veins are joined by a longitudinal vessel called the ascending lumbar vein. On either side of the lumbar vertebrae there may be one or two ascending lumbar veins. The right ascending lumbar vein becomes the azygos vein as it enters the thorax, and the left ascending lumbar vein is continuous with hemiazygos chain.^13^ If the inferior caudal vein (CVC/IVC) is occluded, blood from the lower extremities may reach the heart through the paravertebral and azygos systems. If the inferior vena cava is congenitally absent, the same avenues is utilized.^13^ Normally in species such as the dog the azygos vein empties into the cranial vena cava and then into the right atrium.^4^ The presence of the hemiazygos vein is variable, and when present it is located left to the aorta, communicating the azygos vein with the CVC.^4^ In the present case, the enlarge hemiazygos vein was the major drainage channel from the abdomen, and it emptied into the coronary sinus that opens into the right atrium, and similar findings have been reported in the human literature.^13^

Larger azygos/hemiazygos vein can be misinterpreted as an aortic dissection or mediastinal mass.^5^ Moreover, the enlarged azygos/hemiazygos arch may be mistaken for a right paratracheal adenopathy on the chest radiography.^14^ The authors were able to make the correct diagnosis by means of CT scan, which has been accepted as a valuable modality for demonstrating IVC anomalies.^15^ Although most of IVC interruption with azygos/hemiazygos continuation is usually an asymptomatic malformation, a dozen cases of deep vein thrombosis have been causally linked to IVC anomaly in the English literature.^5^,^9^,^15^,^16^ Theoretically, this anomaly may predispose to venous thrombosis because an inadequate blood return through the collaterals may increase the venous pressure in the veins of the leg, thereby favoring venous stasis.^15^,^16^,^17^

The absence of CVC/IVC may lead to procedural difficulties during femoral vein catheter advancement^9^, IVC filter placement^18^, temporary pacing through the transfemoral route^1^, electrophysiology studies^19^,^20^, and cardiopulmonary bypass surgery.^21^ Awareness of the existence of these anomalies before femoral vein catheter advancement or other procedure through femoral vein would avoid unnecessary injury or undue delay. The recognition of this congenital venous anomaly (CVC/IVC interruption with azygos/hemiazygos continuation) is important for interventional radiologist and cardiologist, especially for conditions such as venous thromboembolism, IVC filter placement, transcatheter closure of the ASD, ventricular septal defect (VSD) patent foramen ovale (PFO) shunt^22^, or pacing and electrophysiology, cardiopulmonary bypass surgery, and palliative systemic venous-pulmonary artery shunt surgery.
